# Effectiveness of Specialized Nutritious Foods and Social and Behavior Change Communication Interventions to Prevent Stunting among Children in Badakhshan, Afghanistan: Protocol for a Quasi-Experimental Study

**DOI:** 10.3390/mps4030055

**Published:** 2021-08-13

**Authors:** Sajid Bashir Soofi, Gul Nawaz Khan, Shabina Ariff, Arjumand Rizvi, Mohammad Asif Hussainyar, Cecilia Garzon, Martin Ahimbisibwe, Rafiullah Sadeed, Ahmad Reshad

**Affiliations:** 1 Department of Paediatrics and Child Health, Aga Khan University, Karachi 74800, Pakistan; shabina.ariff@aku.edu; 2 Centre of Excellence in Women and Child Health, Aga Khan University, Karachi 74800, Pakistan; gul.nawaz@aku.edu (G.N.K.); arjumand.rizvi@aku.edu (A.R.); 3 Aga Khan University Academic Projects, Kabul 1006, Afghanistan; asif.hussainyar@aku.edu; 4 World Food Programme, Kabul 1003, Afghanistan; cecilia.garzon@wfp.org (C.G.); martin.ahimbisibwe@wfp.org (M.A.); 5 Aga Khan Foundation, Badakhshan 3401, Afghanistan; rafi.sadeed@akdn.org; 6 Aga Khan Health Services, Badakhshan 3402, Afghanistan; ahmad.reshad@akdn.org

**Keywords:** stunting, specialized nutritious foods, social and behavior change communication

## Abstract

Stunting predominantly occurs during the first 1000 days of life and continues to the age of five years. We will aim to assess the effectiveness of specialized nutritious foods (SNF)and social and behavior change communication (SBCC) strategies during the first 1000 days of life to prevent stunting among children in two rural districts of Badakhshan, Afghanistan. This will be a quasi-experimental pre-post study with the control group utilizing qualitative and quantitative methods. Before launching the program, formative research will be conducted on the acceptability, appropriate use and SBCC strategies needed to support the introduction of intervention package. Repeated cross-sectional baseline and endline surveys will be conducted in both the intervention and the control districts. After the formative research and baseline household survey, an intervention focusing on the provision of SNF, targeting pregnant and lactating women and children 6–23 months, and SBCC strategies will be implemented for at least 12 months. The primary outcome will be a reduction in the prevalence of stunting among children under two years in the intervention group compared to the control group. We will aim to compare the intervention and control group between the pre- and post-intervention assessments to isolate the effect of the intervention by difference-in-differences estimates. The program monitoring and evaluation component will examine the quality of implementation, acceptability of intervention, identification of potential barriers and to learn how to enhance the program’s effectiveness through ongoing operational improvements. The results will be beneficial to design interventions to prevent stunting within Afghanistan and other low–middle-income countries.

**Trial Registration:** Clinicaltrials.gov NCT04581993 [Registered: 8 October 2020].

## 1. Introduction

Malnutrition is an indicator of social and political instability as it represents a multifaceted problem linked to poverty, food insecurity and poor hygiene and health [1]. Optimal infant and young child feeding (IYCF) practices including an early initiation of breastfeeding, exclusive breastfeeding for 6 months and the introduction of micronutrient-rich and age-appropriate complementary foods from 6 months are particularly important for ensuring the healthy growth and development of young children [2,3,4,5,6]. In 2020, an estimated 149 million children worldwide under five were stunted and 45 million were wasted [7]. The prevalence of undernutrition is significantly higher among unstable and conflict-affected settings [8]. Almost all the countries ranked within the highest 10% of the Global Hunger Index [9] and with the highest stunting rates are classified as unstable or conflict affected [10]. Afghanistan is the only country in the South Asia region in a conflict-affected and fragile situation [10,11]. Afghanistan also ranks 8th on the Global Hunger Index, 14th for stunting rate and 42nd of 45 countries by the Hunger and Nutrition Commitment Index on the country’s government’s political commitment to tackling hunger and malnutrition [9,12,13]. There is a growing evidence base of effective high impact interventions to reduce preventable malnutrition during the first 1000 days of life, between pregnancy and 24 months of age [1]. These include nutrition-sensitive and nutrition-specific interventions.

Several complementary feeding strategies, including the use of specialized nutritious foods (SNF), have been shown to improve growth outcomes and reduce child stunting in food-insecure settings [14,15,16]. However, these findings show either modest improvements or mixed evidence, with declining rates of growth still observed among groups that receive food supplementation [17,18,19,20,21,22,23,24,25,26]. The evidence suggests that with thoughtful formative research and planning, behavior change communication (BCC) interventions can in fact positively improve infant and young child feeding (IYCF) practices, nutritional status and growth [27]. Current studies on the impact evaluations of large-scale BCC interventions to improve IYCF practices in several countries have shown that intensive interpersonal counseling combined with mass media and community mobilization activities have positive impacts on breastfeeding [28,29] and complementary feeding practices [28,30,31].

Worldwide, although investments have been made in implementing nutrition programs and generating robust evidence, peer-reviewed data are limited and program knowledge, often within the grey literature, is not widely shared or transferred. A 2018 series on nutrition in South Asia included two reviews, one on maternal nutrition interventions and another on optimal breastfeeding interventions. No studies from Afghanistan were eligible for these reviews to assess the effectiveness of program approaches to improve the coverage of maternal nutrition interventions and predictors of optimal breastfeeding among children 0–23 months [32,33]. However, there is a need to assess the effectiveness of SNF and social and behavior change communication (SBCC) strategies from Afghanistan to generate evidence for policy development and program implementation in the country and to be scaled-up in other fragile settings facing similar challenges.

### 1.1. Description of Stunting Prevention Programme

As part of the Country Strategic Plan, the World Food Program (WFP) planned to implement a stunting prevention program in collaboration with the Ministry of Public Health (MoPH) through its Public Nutrition Department, Afghanistan in two selected districts of Badakhshan with stunting rates (HAZ < −2SD) of >45% [34]. These two districts were considered based on high stunting prevalence, access to beneficiary communities, the existence of implementing partners providing complementary health/nutrition and security access to implementation sites. The program will emphasize an appropriate nutrition support in the ‘1000 days’ window of opportunity with special focus on proven effective nutrition interventions such as appropriate breast feeding, complementary feeding, micronutrient supplementation, malnutrition treatment and prevention and hand washing. This will, therefore, take the form of an integrated approach ensuring the targeted beneficiaries are supported to access nutrition assistance as well as other complimentary interventions provided in-kind by study partners and will be promoted through SBCC. The program will target pregnant and lactating women (PLW) and children aged 6–23 months, but will also engage fathers, grandfathers, grandmothers and community players that influence maternal, infant and young child nutrition (MIYCN).

The program will include monthly distributions of SNF for PLW and children aged 6–23 months and counselling for their mothers and caretakers on the appropriate use of SNF. All the trainings and SBCC messages will be developed based on formative research on the knowledge, attitudes, practices and barriers to optimal IYCF practices. During the project implementation, emphasis will be placed on the adoption of the locally formulated complementary foods as well as the application of knowledge and practices promoted through the SBCC activities as part of the exit strategy and ensuring a long-lasting impact of the project.

The program will be implemented within the health delivery structure involving monthly implementation and delivery of a package of interventions with the support of the MoPH and Aga Khan Health Services Afghanistan, who will facilitate implementation via the health facilities and at a community level through community health workers/volunteers by Aga Khan Foundation, Afghanistan. To be embedded in the health delivery system, the basic package of health services facilities will be the reference sites to support the provision of complementary services. Community based mechanisms such as mobile health teams (MHTs) and community health workers (CHWs) will support beneficiary access and link with health/nutrition services. MHTs will specifically be engaged where applicable to move services closer to beneficiaries at a community level. MHTs will work through CHWs and other community resource persons to mobilize targeted beneficiaries (PLW/caregivers) for services. Beneficiary support groups such as mother care groups will be formed and will be the smallest unit for delivery of the intervention package.

The stunting prevention program will focus on the 1000 days’ window of opportunity to promote appropriate IYCF practices among children in the Badakhshan Province of Afghanistan.

### 1.2. Intervention Package and Delivery Mechanism

The interventions will focus on the provision of SNF to eligible beneficiaries and SBCC messaging delivered through existing health systems, mobile health teams and CHWs at community levels. All children aged between 6–23 months will receive locally produced lipid-based nutrient supplement–medium quantity (LNS-MQ) and PLW will receive Super Cereal during the study.

*Super Cereal—Wheat Soya Blend with Sugar:* Super Cereal is prepared from heat-treated wheat and whole soya beans, sugar, vitamins and minerals by the WFP. This product is pre-packed and available in 1.5 kg packets. The WFP food safety and quality control unit is responsible for the quality assurance of the product during the program period. A monthly ration of 7.5 kg of Super Cereal (250 g per day) will be provided to pregnant women during pregnancy and to lactating mothers for the first 6 months [35].

*Lipid-based Nutrient Supplement–Medium Quantity (LNS-MQ):* LNS-MQ or Wawa Mum is made with heat-treated oil seeds, pulses, cereals, milk powder, sugar, vegetable oils, vitamins and minerals. Wawa Mum is manufactured from ingredients that are fresh, of good quality and free of foreign materials, infestations and substances hazardous to health. It does not contain any ingredients of animal origin, except dairy products. A daily sachet of 50 g of Wawa Mum will provide 255 Kcal. A monthly ration of 30 sachets will be provided to children during 6–23 months of age. The WFP food safety and quality control unit is responsible for the quality assurance of the product during the program period [36].

**Figure 1:**
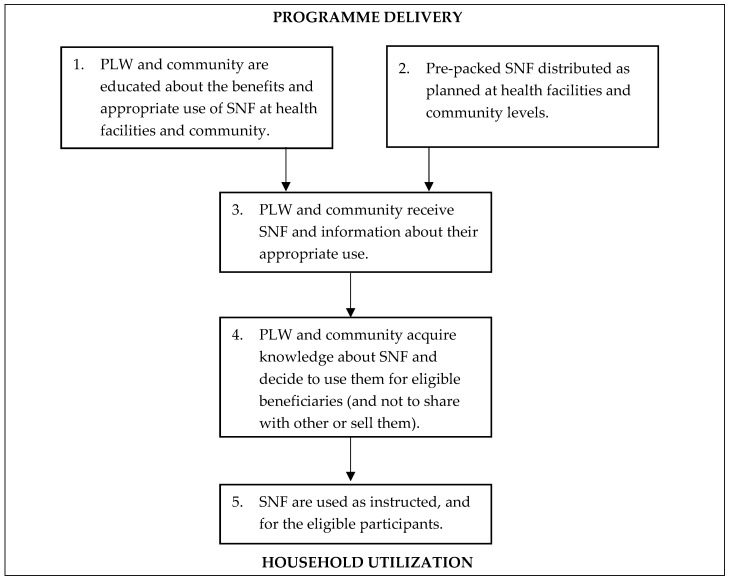
Program theory of process pathways for the intervention package.

*SBCC:* The SBCC strategy will be determined by a literature review and formative research. A cascade training approach will be followed, involving an initial training of master trainers followed by training for each selected health facility staff and community health workers. The minimum qualification for master trainers will be 14th grade education with 2–3 years of experience in maternal and child nutrition. The training will provide information on IYCF practices, child and maternal nutrition and nutritional supplements. Lead mothers will work as facilitators with support from CHWs. Information and demo guides including IEC materials will be used, highlighting selected key messages based on the nutrition issues in the targeted community. To create awareness in the community and at the household level, female and male support groups will be formed and/or strengthened in the catchment areas of CHWs. Female CHWs will form female health committees and male CHWs will form male health committees in their catchment areas. Meetings of both groups will be arranged with the assistance of the community support group and health workers on a monthly basis for the dissemination of SBCC messages related to IYCF practices, child and maternal nutrition and the use of nutritional supplements. Separate sessions on health education for pregnant women, lactating mothers, mothers-in-law, fathers and fathers-in-law will be conducted through the community support groups in the intervention areas using educational materials (flip charts) on IYCF practices and maternal and child nutrition. Considering their important role in decision-making, male members of the family will be encouraged to actively participate in these sessions. The AKU monitoring and evaluation team will assess and monitor the SBCC messages using monthly post distribution visits at health facility and household levels (Table 1, Figure 1).

**Key SBCC Messages:**

1. Eat at least five different food groups every day (grains, white roots and tubers and plantains; pulses (beans, peas and lentils); nuts and seeds; dairy products (milk, yogurt, cheese); meat, poultry and fish; eggs; dark green leafy vegetables; vitamin A-rich and other fruits and vegetables) during pregnancy and the lactation period.
2. Use 250 g (two cups) of Super Cereal every day during pregnancy and the first 6 months of lactation and avoid sharing with family members.
3. Initiate breastmilk to newborn within 1 h of birth, continue exclusive breastfeeding until 6 months of age and introduce nutritionally adequate and safe complementary (semi-solid and solid) foods at 6 months together with continued breastfeeding up to 2 years of age or beyond.
4. Give your child a variety of food groups every day (grains, roots and tubers; legumes and nuts; dairy products (milk, yogurt, cheese); meat, fish, poultry, liver/organ meats; eggs; Vitamin A-rich and other fruits and vegetables) during 6–24 months of age. Do this a minimum of two times for breastfed infants aged 6–8 months, three times for breastfed children aged 9–23 months and four times for non-breastfed children aged 6–23 months.
5. Give one sachet of LNS to your child every day from 6–24 months and avoid sharing with other children.
6. Wash your hands with soap before preparing food, before eating, before feeding a child, after handling feces/diapers or using the latrine and keep the cooking utensils clean to prevent frequent illnesses.

### 1.3. Primary Outcome

The impact of nutritional supplementation during the ‘1000 days’ on the reduction in the prevalence of stunting (LAZ < −2 SD) among children under two years of age in the intervention areas compared to control areas.

### 1.4. Secondary Outcomes

- Impact on low birth weight (birth weight < 2500 g) by improving maternal nutrition.
- Impact on reduction in the prevalence of wasting (WLZ < −2 SD) and underweight (WAZ < −2 SD) in children under two years of age.
- Improvement in IYCF practices.
- Improvement in minimum acceptable diet and minimum dietary diversity among PLW.
- Improvements in care-givers’ knowledge, attitudes and practices related to infant and young child feeding.
- Improvement in mean hemoglobin concentrations in children and PLW.
- Improvement in maternal BMI z-scores.
- Formulation and adoption of local recipes for complementary feeding of children aged 6–23 months using locally available nutritious foods.

## 2. Experimental Design

### 2.1. Study Design

A quasi-experimental pre-post study design with a control group will be used to evaluate the study outcomes. We propose to compare the intervention and control group between the pre- and post-intervention assessments to isolate the effect of the intervention by difference-in-differences estimates. Repeated cross-sectional baseline and endline surveys will be conducted in both the intervention and control districts. The intervention districts in province Badakhshan will be subjected to a robust implementation and close monitoring of the SNF provision, together with SBCC activities delivered through health facility staff, CHWs and mother care groups, while the control districts in province Takhar will receive the routine standard of care available in the area.

Before launching the intervention, formative research will be conducted on the acceptability and appropriate use of nutritional supplements and to help inform the SBCC strategies related to supplements, maternal diet during pregnancy and lactation and IYCF practices. A literature review will be conducted to adapt the relevant themes and existing national guidelines on maternal, infant and young child nutrition (MIYCN) to the local context of Afghanistan. Qualitative methods will be used for formative research including focus group discussions (FGDs) and key informant interviews (KIIs) with target population and relevant stakeholders and direct observations (DOs) for most common household foods recipes for the PLW and children aged 6–23 months.

Program monitoring, evaluation and learning will be conducted to examine the processes including the quality of implementation, the acceptability of the program components, the identification of potential barriers and to learn how to enhance the program’s effectiveness through ongoing operational improvements. This will be conducted by the AKU and study partners on a monthly basis to assess the distribution of supplements, the number of mother groups formed, the key SBCC messages delivered, food demonstrations, the development of local recipes, the monthly mother group meetings, the CHW meetings held on various SBCC activities and the identification of model families (Figure 1).

### 2.2. Study Area

Afghanistan has an estimated population of 32.9 million, consisting of 34 provinces and 398 districts in 2019. About 71% of the population lives in rural settings [37]. Badakhshan province is located in the farthest north-eastern part of the country bordered with Tajikistan, Pakistan and China. This study will be conducted in two districts in Badakhshan (Shuhada and Shari Buzurg), while the control will be in the two districts Rustaq and Worsaj in Takhar province. Takhar province is selected as the control location for the study because these two districts are almost similar in terms of accessibility, socio-economic conditions, food consumption practices, geography and ethnicity. On the other hand, Badakhshan is selected as an intervention area because the selected districts are under the coverage of implementing partners AKHS and AKF and an additional layer of close monitoring by the AKU, the WFP and the MoPH will also be possible. The Badakhshan province is selected for this study because the maternal child health characteristics are poor, but the province is generally less affected by the political instability affecting the rest of the country.

## 3. Procedures

### 3.1. Development of Data Collection Instruments

In collaboration with study partners and local staff, the Aga Khan University will develop topic guides on nutritional intake for mothers during pregnancy and lactation, IYCF practices and the acceptability of and perceptions toward the Super Cereal and LNS-MQ among all participants. We will explore the contextual elements such as interaction with the health care system, livelihoods and other aspects of local culture. We will develop semi-structured, KII [38] and FGD [39] guides with major questions, sub questions and probes in English and will translate into local language, e.g., Dari. To ensure the accuracy of translation, these guides will also be back translated from Dari to English by core study team members [40]. A direct observation guide will also be developed to observe local household food recipes for PLW and children 6–23 months of age by the field researchers [38].

The baseline/endline household survey questionnaire will be developed and used to collect data on household size, socio-demographic characteristics, hand washing, maternal and child anthropometric measurements, maternal and child hemoglobin measurements, the minimum dietary diversity for pregnant and lactating women, antenatal and postnatal care, IYCF practices, maternal knowledge about IYCF, child immunization, morbidity and care seeking, exposure to other interventions and the household food insecurity access scale.

### 3.2. Hiring and Training of Research Staff

We will hire and train a local team of four field researchers (two male and two female), fluent in English and in the local language, i.e., Dari for formative research. The minimum qualification for field research will be 14th grade education with 2–3 years of experience in qualitative research. The training will include principles of qualitative research, in-depth interviewing techniques and FGD and DO techniques. The team will be trained in five days. After training, the field researchers will have three days of practice in the field to test the interview guides for understanding with participants of similar socioeconomic characteristics who will not participate in real data collection. The interview guides will be adjusted if needed after field testing. During field practice, each field researcher will conduct two KIIs, two FGDs and two DOs. The data from the KIIs and the FGDs will be digitally recorded. The recorded data will be transcribed verbatim in the local language and then will be translated verbatim into English. During the practice, field researchers will also transcribe and translate verbatim [38,41] (from the local language into English) the content of the KII and the FGD and write their DO reports in English.

A total of eight data collection teams will be hired locally from Badakhshan and Takhar for the baseline and endline survey. The minimum qualification for data collectors and team leaders will be 12th and 14th grade education, respectively. Each team will consist of two female data collectors and one male team leader. The household survey teams will receive five days of hands-on training on study objectives, informed consent, methods, data collection tools, anthropometric measurements and spot hemoglobin testing. All the questionnaires will be pre-tested in the field and changes will be incorporated accordingly before the actual data collection. The questionnaires will be developed in English and will be translated into Dari (the local language) and then back translated into English by an independent person to compare the translated document with the original for accuracy and quality. A one-day field test will be carried out before initiating field work. As part of the training, all field staff will be trained on anthropometric measurements with additional days for team measurers to continue to refine their skills. The training will include both in class explanations and exercises with field practice. A study manual will be provided to each team leader, which will include instructions, methodology and sampling strategy.

### 3.3. Sample Size and Sampling for Formative Research

To define the sample size, the concept of data saturation will be used. Data saturation is defined as the point where the data being collected do not provide new insights into the research questions [38,41,42]. The number of participants required to reach data saturation varies depending on the objectives of the study and the complexity of the topics being explored. However, we are planning to conduct four FGDs with pregnant and four with lactating women. We will also conduct two FGDs with mothers-in-law and two with the fathers of children under 24 months of age. Eight KIIs will be conducted with CHWs, four with health care providers, four with focal persons of study partners and four with community leaders. A total of fourteen DOs will be held in households for observation of the most common recipes for PLW and children 6–24 months of age. Data will be collected in the catchment areas of seven intervention group health facilities. A purposive sampling method will be used to select the participants and will ensure representation of the different health facilities and participants.

### 3.4. Sample Size for Programme Evaluation Surveys

A total of 2912 children <2 years with their mothers (1456 per group per survey) were estimated to provide reasonable precision (80% power, 95% confidence interval, design effect of 1.5 and response rate of 95%) for baseline and endline surveys based on a 13% reduction in the prevalence of stunting in children under two years old in the intervention areas, as compared in the control areas at endline.

To calculate the sample sizes for anemia (1125 mothers and 810 children per group per survey) with 80% power, a 95% confidence interval, a design effect of 1.5 and a response rate of 95% were estimated to detect a relative 20 and 13% decline in the prevalence of anemia among mothers and in children at endline, respectively.

All pregnant and lactating mothers with children under two years of age will be eligible for baseline and endline surveys. Eligible participants will be identified through two levels.

1. At health facilities, a list of pregnant women who accessed ANC services and breastfeeding mothers who accessed PNC and childcare services will be prepared.
2. At the community level, a list of PLW and children will be prepared through CHWs. These will include those PLW and children who were not captured at the health facility level.

The baseline and endline surveys will follow a two-stage cluster sampling design. In the first stage, 30 clusters will be randomly selected from each intervention and control area (60 total) using probability proportional to the population size (PPS) sampling design. The data collection teams will prepare a list of all the PLW with children under two years old in each selected cluster and required households from each cluster will be randomly selected for the survey. There will be no replacement in case of refusal. The same sampling design will be used at endline.

### 3.5. Data Collection

We will use qualitative methods including FGDs, KIIs and DOs to gather detailed information on maternal and child nutrition practices, and the influence of local cultural beliefs related to these practices. All the focus group discussions and interviews will be audio recorded after obtaining oral consent from the participants. The field researchers will use an observation guide to perform their DO work. While observing, the researchers will take notes of key things that they see. Once the observation is finished, the field researchers will immediately write expanded notes of their observations and write a report. DOs will be carried out in the households of PLW. Each of these observations will focus on the preparation and eating habits of most common food recipes for PLW and children aged 6–24 months throughout the day and on interactions between family members. In the field notes, the field researchers will write their own reflections and impressions of the information they collected through the KIIs, FGD and DO. The core study team members (study coordinator and study manager) will supervise the data collection during practice and during the data collection. Supervision will be carried out by reading the transcripts line-by-line and giving detailed feedback on each transcript from KII, FGD, as well as the DO reports. The study investigators will have close communication with the field researchers by email, other messaging systems and by Skype meetings.

Baseline and endline data will be collected on paper forms and then will be entered on tablets. A customized application will be developed using Java on SQLite backend for data storage. The key features of the data collection application will include access control, onscreen consistency and range checks, onscreen tips and quick reports. Range and consistency checks as well as skip patterns will be built into the program to minimize the entry of erroneous data. Special arrangements will be made to enforce the referential integrity of the database to ensure that all data tables are related to each other.

Two trained staff will measure anthropometric measurements. The first measurer will measure and record each anthropometric measurement without revealing the values obtained to the second measurer. The second measurer will then independently repeat the same measurements. Each measurer will record their own values independently with no knowledge of the values recorded by the other measurer. After collecting the data, the two measurers will compare their measurements to ensure that the differences between their measurements fall within the standard maximum allowed differences (7 mm for length and 50 g for weight). Any pair of measurements falling outside the maximum allowed differences will be repeated by both measurers and will be entered on the recording sheet. If this second pair of measurement values again exceed the standard limits for that measurement, the measurers will repeat the measurement for a third and final time.

Mother and child weight will be assessed using a calibrated balance allowing double weighing (mother–child) and an automatic deduction of the mother’s weight to obtain the child’s weight. An SECA brand weight machine will be used for weight measurement with an accuracy of 50 g. The children’s length will be measured using an SECA length board with an accuracy of 0·1 cm. Mother and child hemoglobin levels will be tested using HemoCue Hb 201 analyzers.

At the endline survey, mothers will be asked about the exposure to specific components of the intervention package, such as familiarity with their health facility, CHWs, participation in individual and group counseling sessions and awareness of SBCC messages on maternal diet and IYCF. Maternal dietary diversity will be assessed by Minimum Dietary Diversity for Women and IYCF practices using the WHO IYCF module. Extensive information will also be collected on the use of supplements, including how it was consumed (mixed or eaten alone), if the mother and child liked the Super Cereal and LNS, any changes (both positive and negative) since the mother and child started consuming these supplements and how many monthly batches had been collected from the nearest health facility or from mobile teams or CHWs. The mothers’ reported recall along with supplements’ distribution data will be collected to assess the compliance of supplements.

### 3.6. Programme Monitoring, Evaluation and Learning

Program monitoring data will be collected on a monthly basis for process evaluation and learning. The expected utility of this process of monitoring data will be to assess the distribution of supplements, the number of mother groups formed, the key SBCC messages delivered, food demonstrations, the development of local recipes, monthly mother group meetings, CHW meetings held on various SBCC activities and the identification of model families. The data will be collected through monitoring the checklists from study participants, program staff and key stakeholders related to the delivery of the intervention.

### 3.7. Data Analysis Plan

The qualitative data will be analyzed using Framework Analysis developed by Ritchie and Spencer in 1994 [43]. Since the current formative research has specific questions related to food habits and IYCF practices that will help to design the SBCC strategy. The recordings of FGDs and KIIs will be translated into English and supplemented by field notes and informal observation throughout the fieldwork. Two researchers will read through the transcripts and field notes repeatedly to identify emerging themes and will agree upon a preliminary coding framework. A third researcher will look independently at a subset of transcripts to verify the themes in the original framework and identify additional ones. A coding framework will be agreed, and the remaining transcripts will be read and coded by the two researchers. The framework will use our main topics of interest, nutrition during pregnancy and post-partum, breastfeeding and complementary feeding practices, and sub-themes will be identified within these broader categories. In addition, we will identify important underlying themes related to the socio-economic context, which ran through each of the categories mentioned above.

The baseline and endline analysis will be designed to provide estimates of key indicators at intervention and control levels. Initial analysis will include examining the frequency distribution of all the variables to identify possible errors. The final analyses will be performed after data cleaning and satisfactory quality assurance. Descriptive statistics for the subjects will be estimated and reported as means (±SD), medians, ranges and frequencies as appropriate. The anthropometric measurements, together with the age and gender of the children, will be used to calculate the weight-for-age, height-for-age and weight-for-height z-scores. The prevalence of malnutrition in its different forms (underweight, wasting and stunting) will be calculated using the z-score cut-off point of <2 SD using the WHO growth standards 2006. BMI will be calculated by dividing their weight in kilograms by the square of their height in meters as Underweight (<18.0 kg/m^2^), Normal (18.0–24.9 kg/m^2^), Overweight (25–29.9 kg/m^2^) and Obese (≥30 kg/m^2^. Anemia will be defined as hemoglobin levels <11gm/dl for children aged 6–23 months and <12gm/dL for PLW. All analysis will be performed on Stata version-15.

## 4. Results

The primary study finding will be the impact of intervention on the prevalence of stunting among children under two years old in the intervention group compared to the control group. Secondary findings will be the prevalence of low birth weight in newborns and the prevalence of wasting, underweight, anemia and IYCF practices among children under two years of age. Furthermore, we will also report the minimum dietary diversity, anemia and nutritional status among PLW. We will aim to compare the results of the intervention and the control group between the pre- and post-intervention assessments to isolate the effect of the intervention by difference-in-differences estimates.

## 5. Discussion

To our knowledge, this will be first study to explore the effects of nutritional supplements and SBCC on a reduction in the prevalence of stunting during the first 1000-days of life in Afghanistan. The study findings will be disseminated through workshops held at provincial, country and international levels. The findings from this study will be disseminated via conference presentations and publications in peer-reviewed journals.

The implications of this project are substantial as the program is aimed to prevent stunting through empowering the targeted communities and strengthening their capacity to adopt appropriate IYCF practices; the feedback on progress will be shared with communities through their monthly mother care group sessions. The results generated from the study will be beneficial to design interventions to prevent stunting within Afghanistan and other similar low–middle-income countries.

## 6. Study Strengths and Limitations

- The study will be among the first to evaluate the effectiveness of SNF targeting pregnant and lactating women and children aged 6–23 months and SBCC strategies on a reduction in the prevalence of stunting among children in Afghanistan.
- Program monitoring and evaluation data will allow us to examine the quality of implementation, the acceptability of intervention, the identification of potential barriers and to learn how to enhance the program’s effectiveness through ongoing operational improvements.
- The cohort of enrolled PLW and children will help to understand the feasibility and adherence to intervention within a community over a 12-month period.
- The quasi-experimental design limits the choice in the selection of study area and the random selection of health facilities.

## Figures and Tables

**Table 1:** Program implementation and SBCC strategy.

Intervention Activities	Frequency	Lead	Implementing Partners	Sources and Mean of Verification
PLW will receive 7.5 kg of super cereal during pregnancy and first 6 months of lactation. Children 6–23 months will receive 30 sachets of LNS-MQ. Anthropometric measurements by AKHS health facility staff.	At enrolment and monthly	WFP, AKU and MoPH	AKHS, AKF 2 Mobile Teams	Recruitment registers Household M&E data Monthly report
Train 80 CHWs on SBCC and intervention activities by AKF.	Once at start of project	WFP, AKU and MoPH	AKF	Training sheet Key SBCC messages Flip charts Monthly report
Train 800 family health action group (FHAG) members in 40 health posts on maternal nutrition, IYCF and key SBCC messages by AKF.	Once at start of project	WFP, AKU and MoPH	AKF	Training sheet Flip charts Key SBCC messages Household M&E data Monthly report
200 SBCC sessions with parents in program villages by AKF (Total 4000 participants/20 per session). SBCC messages delivered by AKHS health facility staff in each visit. SBCC messages delivered by CHWs.	Once at start of project and monthly	WFP, AKU and MoPH	AKF, AKHS	Key SBCC messages Flip charts Household M&E data Monthly report
Train and follow-up of 5000 PLW at community level about the importance of 1000-days, maternal nutrition, IYCF and key SBCC messages (25 PLW/session in program villages).	Once at start of project	WFP, AKU and MoPH	AKF	Key SBCC messages Flip charts Household M&E data Monthly report
200 healthy food preparation and demonstration sessions by AKF in all catchment villages (1 session/village).	Once at start of project	WFP, AKU and MoPH	AKF	Food items and demonstration equipment Key SBCC messages Monthly report
Training for 14 health facility staff of AKHS (2 staff per health facility).	At start and mid of study	WFP, AKU and MoPH	AKHS	Pre-post-test Midterm report

## Data Availability

Not applicable.
